# Pembrolizumab induced-C3 glomerulonephritis and RBC cast nephropathy: a case report

**DOI:** 10.1186/s12882-023-03202-5

**Published:** 2023-05-24

**Authors:** Zhi Yang, Huan Xu, Shenju Gou, Hongyan Wu, Zhangxue Hu

**Affiliations:** 1 Department of Nephrology, West China Hospital, Sichuan University, 37# Guoxue Alley, Wuhou District, Chengdu, 610041 Sichuan Province China; 2 Department of Pathology, West China Hospital, Sichuan University, 37# Guoxue Alley, Wuhou District, Chengdu, 610041 Sichuan Province China

**Keywords:** C3 glomerulonephritis, Immunotherapy, PD-1 inhibitors, Pembrolizumab, RBC cast nephropathy

## Abstract

**Background:**

Immune checkpoint inhibitors (ICIs) are increasingly being used in the treatment of several cancers. Pembrolizumab is an anti-programmed cell death-1 (anti-PD-1) monoclonal antibody that is approved for the treatment of metastatic non-small cell lung cancer (NSCLC). Pembrolizumab-associated renal toxicity is relatively rare, even in pembrolizumab-associated glomerulonephritis. In this study, we report a rare case of pembrolizumab-induced C3 glomerulonephritis (C3GN) and RBC cast nephropathy.

**Case presentation:**

A 68-year-old man with NSCLC was receiving treatment with pembrolizumab. After 19 cycles of pembrolizumab therapy, he presented with gross hematuria, severe lower-limb edema and oliguria. Laboratory tests revealed hypoalbuminemia, increased serum creatinine and low serum C3 level. Renal biopsy revealed a typical membranoproliferative glomerulonephritis accompanied by remarkable RBC casts in tubular cavities and tubulointerstitial infiltration of CD8-positive lymphocytes. Based on C3-only immunofluorescence deposit on glomeruli, a diagnosis of C3GN was made. Pembrolizumab was considered the cause of C3GN. Pembrolizumab was discontinued immediately, and 60 mg/day of prednisone was initiated. One dose of cyclophosphamide (400 mg, IV) was also administered. Upon treatment, his symptoms improved rapidly and serum creatinine decreased a lot. However, the patient became dialysis dependent eventually.

**Conclusion:**

This is the first case of C3GN with RBC cast nephropathy caused by ICIs. This rare case caused by the prolonged use of pembrolizumab further strengthens the relationship between ICIs and C3GN. Thus, periodic evaluation of urine and renal function is recommended in patients receiving pembrolizumab and other ICIs.

## Introduction

Immune checkpoint inhibitors (ICIs) are increasingly used in the treatment of several malignancies. Pembrolizumab is a humanized monoclonal antibody designed to directly block the interaction between programmed cell death protein 1 (PD-1) and its ligands, leading to T-cell stimulation and anti-tumor response, and it has been successfully used in the treatment of melanoma, lung cancer, renal cancer, and hematological malignancies [1]. However, it may also alter self-tolerance and cause ICI-related adverse effects, i.e., side effects caused by excessive and nonspecific immunologic activities. Compared with ICI-associated colitis, pneumonitis, skin toxicities, or endocrinopathies, kidney adverse effects are relatively rare [2]. ICIs related nephrotoxicity mainly involves acute tubulointerstitial nephritis (AIN). Only a few cases of ICIs related glomerulonephritis have been reported and no RBC cast nephropathy caused by ICIs has been reported. In this study, we report a case of ICI-associated C3 glomerulonephritis (C3GN) and RBC cast nephropathy following treatment with pembrolizumab. The report of this case further broadens the spectrum of ICI-associated GN and provides more evidence of the link between C3GN and ICIs.

## Case report

The patient was a 68-year-old man diagnosed with non-small-cell lung cancer (NSCLC) staged IIIB (T3N2M0) two years ago. The tumor was surgically removed. After surgery, the patient had completed six cycles of chemotherapy (two cycles of pemetrexed plus carboplatin and four courses of paclitaxel plus carboplatin). Pembrolizumab (Keytruda, 200 mg IV, once every 3 weeks) was initiated since the third course of chemotherapy. After 9 months of treatment, the cancer progressed to stage IVB (T4N2M1) with multiple metastases in both lungs confirmed by percutaneous pulmonary biopsy. Then, anlotinib (a multi-targeted tyrosine kinase inhibitor, TKI) was started, with a prescribed dose of 10 mg orally daily, for 2 weeks on/1 week off. During the course of treatment, blood tests and urinalysis were performed before each chemotherapy and immunotherapy session.

After 19 cycles of pembrolizumab therapy, he abruptly presented with macroscopic hematuria, heavy lower-limb edema and oliguria with chest tightness and cough. Workup revealed a sharp rise in the serum creatinine (sCr) level to 1.82 mg/dL from the baseline level of 0.52 mg/dL and a nephrotic-range proteinuria (spot urinary protein-to-creatinine ratio [UPCR] 9.48 g/g). Accordingly, the patient was diagnosed with acute kidney injury (AKI) and was immediately admitted to the nephrology department for further evaluation and treatment.

Upon admission, his blood pressure, pulse, and temperature were 155/80 mmHg, 83 beats per min, and 36.7 °C, respectively. Bilateral leg edema, chest tightness, cough, bloating, and nausea were noted. A skin rash with itch was found on his left arm. The urine output decreased to 300 mL/day. Felodipine retard tablets (5 mg daily) had been used for more than 10 years to control hypertension. He had no history of diabetes, autoimmune disorders, or kidney diseases. He had no history of taking proton-pump inhibitors, anticoagulants, antibiotics, or nonsteroidal anti-inflammatory drugs. He had not used contrast within 2 weeks. No evidence of kidney or urinary system tumor metastasis was noted.

Regarding laboratory tests, sCr level continued to increase and peaked at 6.13 mg/dL. Serum albumin decreased to 2.8 g/dL. Urinalysis was positive for protein (4 +) and red blood cells (120 cells/high-power field). Urine cultures were negative. Serum C3 and C4 levels were 0.558 (normal range, 0.785–1.52) g/L) and 0.123 (normal range, 0.145–0.360) g/L, respectively. The serum procalcitonin level was 0.65 (normal range, < 0.046) ng/mL. Anti-dsDNA antibody, anti-nuclear antibodies, or extractable nuclear antigens were all negative. No abnormity was found in the serum immunofixation electrophoresis. Ultrasonography revealed that both kidneys were normal and no evidence of kidney or urinary system tumor metastasis was noted. Computed tomography of the chest showed mild inflammation and scattered nodules. More detailed laboratory test results are summarized in Table 1.

**Table 1:**
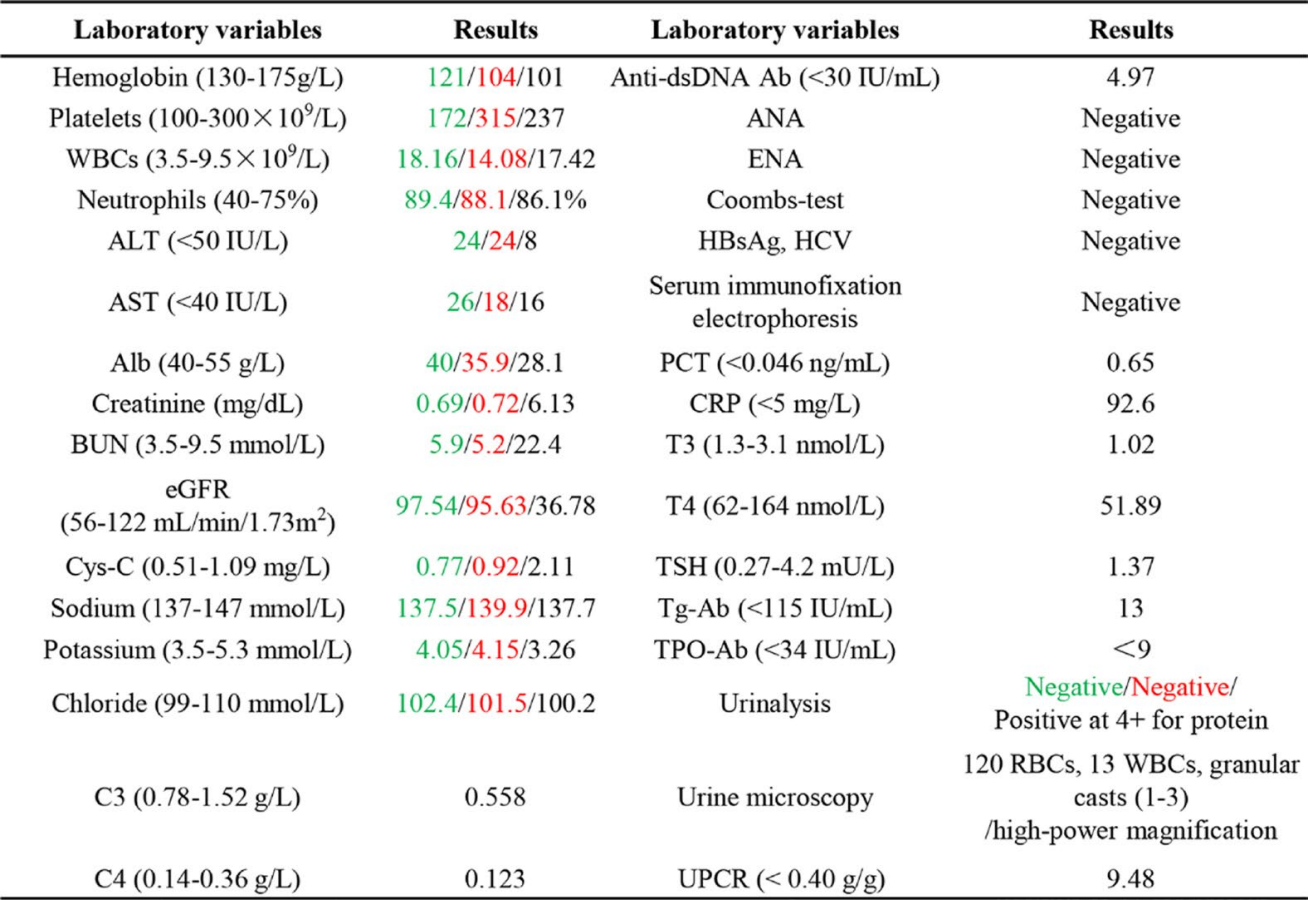
Laboratory data obtained prior to diagnosis of lung cancer (show in green), after the first chemotherapy treatment (show in red) and at the time of kidney biopsy (show in dark)

*WBC* White blood cells, *ALT* Alanine transaminase, *AST* Aspartate transaminase, *BUN* Blood urea nitrogen, *eGFR* Estimated glomerular filtration rate, *C3* Complement 3, *C4* Complement 4, *ANA* Antinuclear antibody, *HBsAg* hepatitis B surface antigen, *HCV* Hepatitis C virus, *PCT* Procalcitonin, *CRP* C-reaction protein, *T3* Triiodothyronine, *T4* Thyroxine, *TSH* Thyroid stimulating hormone, *Tg-Ab* anti-thyroglobulin antibody, *TPO-Ab* Anti-thyroid peroxidase antibody, *UPCR* Urine protein-to-creatinine ratio

**Fig. 1:**
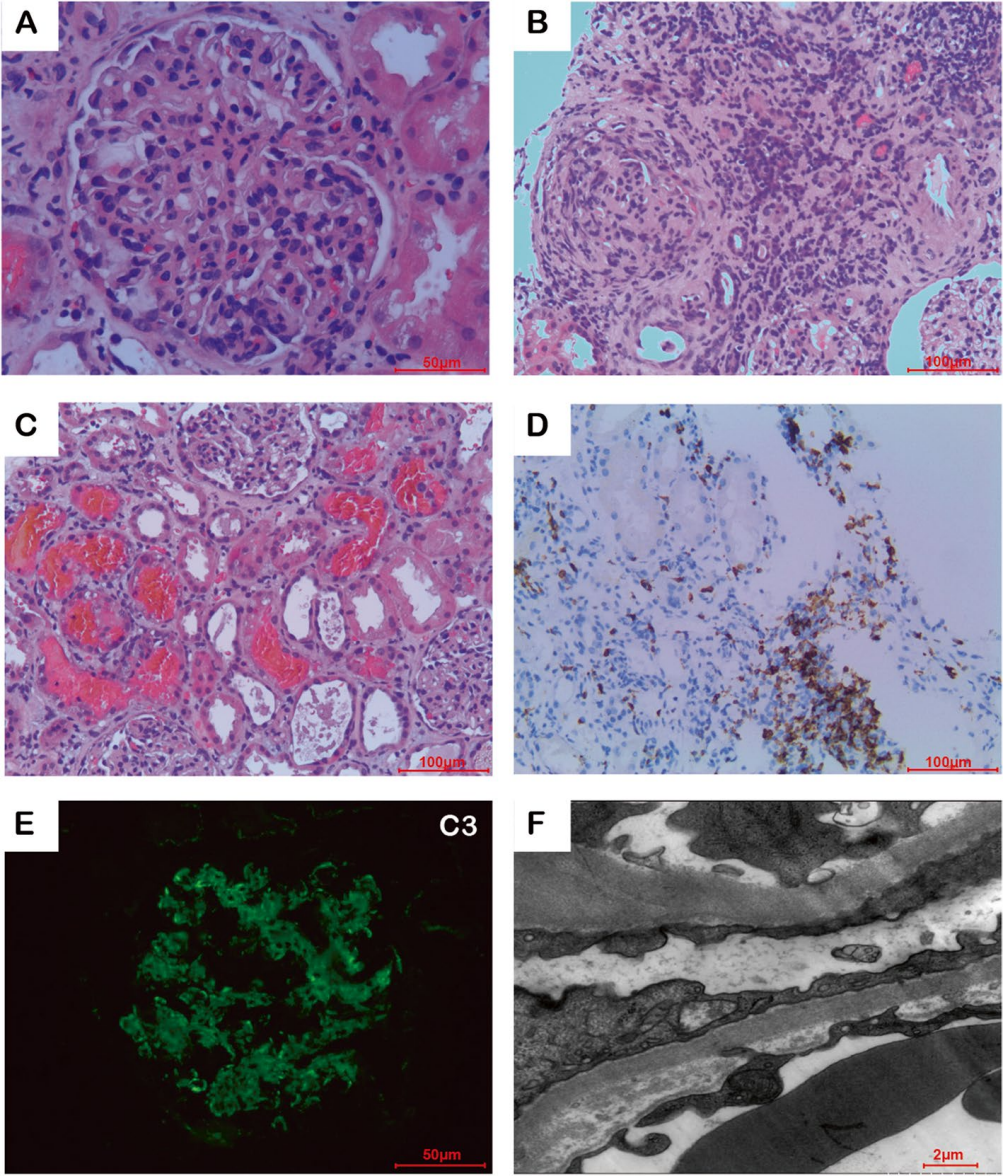
Kidney biopsy performed in the present patient. Panel **A**. Presence of membranoproliferative glomerulonephritis (light microscopy, Hematoxylin–eosin, × 400). Panel **B**. Substantial tubulointerstitial lymphocytes infiltration (Hematoxylin–eosin, × 200). Panel **C**. Tubular cavities were filled with massive RBC casts (Hematoxylin–eosin, × 200). Panel **D**. Most of the infiltrated lymphocytes were positive for CD8 (Anti-CD8 immunohistochemistry, × 200). Panel **E**. C3-only deposits in glomerulus (2 +) (Anti-C3 immunofluorescence, × 200). Panel **F**. Intermediate electron-dense deposits in the sub-endothelial areas (Electron microscopy, × 6000)

A percutaneous kidney biopsy was performed. Light microscopy revealed a typical membranoproliferative glomerulonephritis (MPGN) accompanied by acute tubulointerstitial nephritis (Fig. 1A, B). Remarkable red cell casts were seen in tubular cavities (Fig. 1C). Most of the infiltrated lymphocytes were positive for CD8 (Fig. 1D). The immunofluorescence study showed moderate C3 deposits (2 +) in the mesangium and along the capillary loops, but no significant staining for IgG, IgA, IgM, kappa, lambda, C4, and C1q (Fig. 1E). Ultrastructural analysis showed subendothelial electron-dense immune deposits (Fig. 1F).

**Fig. 2:**
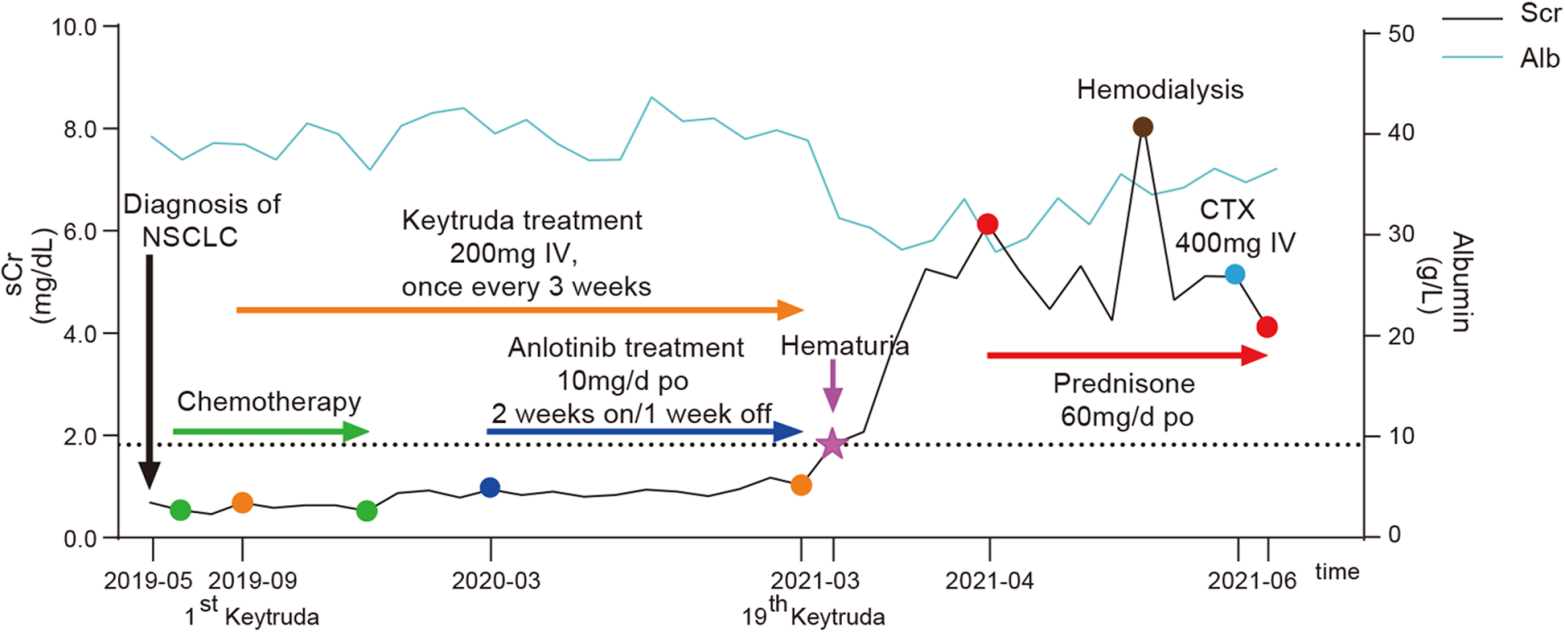
The clinical course of the patient after the initiation of anti-cancer therapy. sCr, serum creatinine. CTX, cyclophosphamide. Alb, albumin. NSCLC, non-small-cell lung cancer. IV, intravenous

Given the clinical manifestation, laboratory examinations, and pathological findings, a diagnosis of C3GN combined with RBC cast nephropathy was made. After ruling out genetic factors or other secondary causes, we considered pembrolizumab as the cause of C3GN. Pembrolizumab was discontinued immediately, while prednisone 60 mg per day and hemodialysis were initiated. One dose of cyclophosphamide (400 mg, IV) was also administered. Combined with supportive treatments, chest tightness, edema and cough improved rapidly. Urine output increased to 600–800 mL/day. Gross hematuria disappeared, and urine red blood cells declined to 9 cells/high-power field. The spot UPCR declined to 3.19 g/g. Serum albumin increased to 3.6 g/dL and sCr level decreased gradually, reaching a nadir of 4.12 mg/dL but still required hemodialysis. The timeline of clinical manifestation and treatment process of the patient was summarized in Fig. 2. With a significant improvement, the patient was discharged from the hospital for outpatient dialysis. Unfortunately, he discontinued hemodialysis after discharge and died 2 weeks later.

## Discussion

ICI-related adverse effects could involve any organ or system, such as the skin, intestine, liver, thyroid, pancreas, pituitary, and kidney [3]. Renal toxicity is generally considered a less common event caused by ICIs, which most commonly manifested as AKI. In an analysis of 3695 patients treated with ICIs (including ipilimumab, nivolumab, and pembrolizumab), the overall incidence of AKI was 2.2% in all patients and 1.4% in the pembrolizumab-treated group [4]. Some patients may also present with proteinuria, hematuria, or pyuria, which indicates glomerular lesions. Acute tubulointerstitial nephritis with infiltration of lymphocytes and plasma cells was the primary pathologic lesions manifesting as acute kidney injury [5]. In recent years, the association between ICIs and glomerular nephritis (GN) has received more attention. Several studies have indicated that ICIs could cause a wide spectrum of GN. A systematic review reported that the top three most frequent types were pauci-immune GN/vasculitis (27%), minimal-change disease (MCD) (20%), and C3GN (11%) [6]. In addition, IgA nephropathy, anti-glomerular basement membrane disease, lupus-like nephritis, membranous nephropathy, amyloid amyloidosis, focal segmental glomerulosclerosis (FSGS), and thrombotic microangiopathy (TMA) have also been reported [6]. The mechanism by which ICIs may cause GN was much less clear. Since different ICI treatments may induce varying renal manifestations and pathological patterns, multiple complex mechanisms may be involved and should be further elucidated.

To the best of our knowledge, this is the sixth case of ICI-associated C3GN [2, 7–10]. The clinical features of the other five cases and the present patient are summarized in Table 2. Among them, 4/6 were associated with pembrolizumab and manifested as AKI; 3/6 presented with concomitant tubular-interstitial injury; 2/6 presented with macroscopic hematuria, and our case is unique for its remarkable red cell casts; 2/6 presented with lower C3 serum level; 3/6 presented with skin rashes; 4/6 responded to ICIs discontinuation and initiation of glucocorticoids.

**Table 2:**
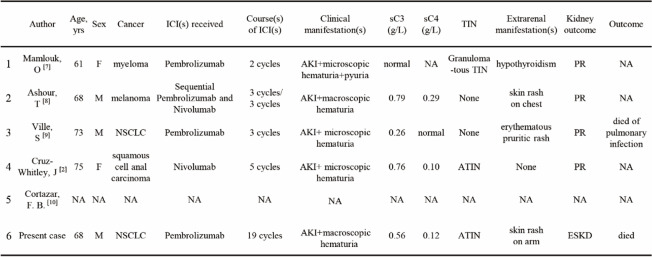
Summary of reported cases of C3GN associated to the use of immune checkpoint inhibitors

*M* Male, *F* Female, *ICI* Immune checkpoint inhibitor, *sC3* Serum C3 level, *sC4* Serum C4 level, *TIN* Tubular-interstitial injury, *ATIN* Acute tubular-interstitial injury, *PR* Partial remission, *ESKD* End-stage kidney disease, *NA* Not acquired

Excessive activation of the complement alternative pathway (AP) is considered the major pathogenic factor leading to C3GN, which typically presents with low C3 serum levels, normal C4 levels and high C5b9 levels [11]. It was assumed that anti-PD-1 therapy may induce the emergence of autoantibodies against C3b promoting C3 deposition via the dysregulation of the complement AP, such as the C3b antibody detected in the case reported by Ville et al. [9]. In this study, the patient presented with low C3 serum level, MPGN-like pattern, C3 deposit in glomeruli, which supported the diagnosis of C3GN. The patient presented skin rashes and AIN which were also ICIs-associated immune injury.

For our case, the abundant infiltration of CD8-positive lymphocytes in interstitium shows an overactivation of T cells, as well as the mechanism by which pembrolizumab works, further strengthening the association between pembrolizumab and AIN. Although anlotinib, a multi-targeted tyrosine kinase inhibitor (TKI) [12], was also a potentially nephrotoxic agent, TMA and podocytopathies, such as MCD and FSGS, were the main presentations of TKI-associated renal toxicity [13]. While, the characterization of the predominant CD8-positive T lymphocyte infiltration, may be caused by the combination of anlotinib and pembrolizumab. Anlotinib is a TKI that targets vascular endothelial growth factor receptor, fibroblast growth factor receptor, platelet-derived growth factor receptors, and c-kit. Recent study showed that CD8-positive T-cell responses are boosted by dual PD-1/VEGFR2 blockade [14]. Thus, Anlotinib might act as a promoter, creating an immune-supportive microenvironment for ICIs [15] and driving the adverse effects of ICIs. However, the mechanism by which ICIs could cause C3GN was still unclear and need to be further explored.

In addition, our case presented with remarkable tubular RBC casts, which had not been reported before. RBC cast nephropathy was usually observed in anticoagulant-related nephropathy, IgA nephropathy and AIN and may cause AKI [16]. Hemoglobin, heme, iron or other molecules released from RBCs could cause direct tubular toxicity. In this patient, remarkable RBC casts were results of ICI-associated C3GN and AIN and aggravated AKI.

The National Comprehensive Cancer Network guidelines for the management of ICI nephrotoxicity are based on the severity of kidney injury [17]. As reported, for patients with ICI-associated GN, most could have either complete (31%) or partial (42%) recovery with ICI discontinuation and corticosteroid treatment, whereas nearly 20% of the cases might progress to end-stage kidney disease (ESKD) [6]. For this patient, although there was an improvement in renal function after a two-week intervention of prednisone and hemodialysis, the renal function worsened again, and we administered cyclophosphamide subsequently. According to the literature [8], patients who experienced grade 3 to 4 ICI-related nephrotoxicity can be treated with immunosuppressants, such as cyclophosphamide, azathioprine, infliximab, and mycophenolate. As the results showed, the patient's serum creatinine levels decreased after the administration of cyclophosphamide. Nevertheless, he still needed hemodialysis when he discharged, maybe due to his concomitant involvement of other organ system. As Cortazar reported, patients who had concomitant extrarenal ICI-related side effects had a lower likelihood of kidney recovery [10]. Thus, when a patient had ICI-associated side effects in more than one organ or system, an immediate diagnosis should be made and positive treatment should be provided.

In conclusion, with the increasing application of ICIs for various malignancies, ICI-associated side effects, especially renal toxicity, should be given more attention. This study presents with a rare case of ICIs-associated C3GN, AIN and RBC cast nephropathy caused by chronic use of pembrolizumab, which further expanded the spectrum of nephrotoxicity of ICIs. As a nephrologist, the recognition of ICI-associated renal side effects is critical, and a kidney biopsy may help in evaluating the underlying cause.

## Data Availability

Most of the clinical data used and analyzed in this case report is presented in this manuscript. More detailed information is available from the corresponding author on reasonable request.

## Abbreviations

ICI: Immune checkpoint inhibitors
C3GN: C3 glomerulonephritis
C3/C4: Complement 3/ complement 4
PD-1: Programmed cell death protein-1
AKI: Acute kidney injury
AIN: Acute tubulointerstitial nephritis
NSCLC: Non-small-cell lung cancer
TKI: Tyrosine kinase inhibitor
sCr: Serum creatinine
UPCR: Urinary protein-to-creatinine ratio
MPGN: Membranoproliferative glomerulonephritis
RBC: Red blood cell
GN: Glomerular nephritis
TMA: Thrombotic microangiopathy
AP: Alternative pathway
TMA: Thrombotic microangiopathy
ESKD: End-stage kidney disease
